# Huperzine A Regulates the Physiological Homeostasis of Amyloid Precursor Protein Proteolysis and Tau Protein Conformation—A Computational and Experimental Investigation

**DOI:** 10.3390/biology13070518

**Published:** 2024-07-12

**Authors:** Suwakon Wongjaikam, Chutikorn Nopparat, Parichart Boontem, Jiraporn Panmanee, Nopporn Thasana, Mayuri Shukla, Piyarat Govitrapong

**Affiliations:** 1 Chulabhorn Graduate Institute, Chulabhorn Royal Academy, Bangkok 10210, Thailand; suwakon.won@mahidol.ac.th (S.W.);; 2 Cell and Animal Model Unit, Institute of Nutrition, Mahidol University, Nakhonpathom 73170, Thailand; 3 Innovative Learning Center, Srinakharinwirot University, Sukhumvit 23, Bangkok 10110, Thailand; 4 Research Center for Neuroscience, Institute of Molecular Biosciences, Mahidol University, Nakhonpathom 73170, Thailand; 5 Program in Chemical Sciences, Chulabhorn Graduate Institute, Chulabhorn Royal Academy, Bangkok 10210, Thailand; 6 Laboratory of Medicinal Chemistry, Chulabhorn Research Institute, Bangkok 10210, Thailand

**Keywords:** Huperzine A, Alzheimer’s disease, amyloid beta, amyloid precursor protein, β-site amyloid precursor protein cleaving enzyme 1, presenilin 1, A disintegrin and metalloproteinase 10, tau

## Abstract

**Simple Summary:**

Alzheimer’s disease is a chronic neurodegenerative disorder that causes brain shrinkage and cellular death. The pathological changes like formation of amyloid plaques and aggregation of hyperphosphorylated tau in the brain begin years before the clinical symptoms appear. Moreover, there has been a drastic increment in the number of people affected by this disease. Unfortunately, there is no cure for Alzheimer’s. In this scenario, natural compounds with potent neuroprotective properties and minimal side effects hold promise. Therefore, the present study investigated the effects of Huperzine A (an alkaloid isolated from the moss *Huperzia serrata*) on the amyloid processing and tau conformations under physiological conditions with an aim of developing a preventive rather than curative approach. The outcome of this study shows that Huperzine A can significantly modulate the amyloid-β and tau processing, suggesting that it could protect the nerve cells, slow cognitive decline, and improve memory. We further suggest that advancement of Huperzine A as a general therapeutic agent might alleviate the socio-economic burden related to the progressive debilitating nature of this disease.

**Abstract:**

The beneficial actions of the natural compound Huperzine A (Hup A) against age-associated learning and memory deficits promote this compound as a nootropic agent. Alzheimer’s disease (AD) pathophysiology is characterized by the accumulation of amyloid beta (Aβ). Toxic Aβ oligomers account for the cognitive dysfunctions much before the pathological lesions are manifested in the brain. In the present study, we investigated the effects of Hup A on amyloid precursor protein (APP) proteolysis in SH-SY5Y neuroblastoma cells. Hup A downregulated the expression of β-site amyloid precursor protein cleaving enzyme 1 (BACE1) and presenilin 1 (PS1) levels but augmented the levels of A disintegrin and metalloproteinase 10 (ADAM10) with significant decrement in the Aβ levels. We herein report for the first time an in silico molecular docking analysis that revealed that Hup A binds to the functionally active site of BACE1. We further analyzed the effect of Hup A on glycogen synthase kinase-3 β (GSK3β) and phosphorylation status of tau. In this scenario, based on the current observations, we propose that Hup A is a potent regulator of APP processing and capable of modulating tau homeostasis under physiological conditions holding immense potential in preventing and treating AD like disorders.

## 1. Introduction

Alzheimer’s disease (AD) is a complex neurodegenerative disorder and is the leading cause of senile dementia, characterized by progressive and irreversible loss of memory [1]. The cumulative aggregation of the extracellular amyloid plaques and formation of intracellular neurofibrillary tangles mark the distinctive pathological features of this disease [2]. Disquietingly, the increasing size of the aging population and AD patients worldwide poses a significant threat to the health care system as a whole due to its chronicity, incurability, and high treatment cost, and hence, it has been conceded as one of the most arduous medical problems [3]. The accumulation of the amyloid β (Aβ) peptide and tau protein abnormalities cause considerable presymptomatic neuronal damage resulting in the failure of effective therapeutics targeting AD. Therefore, the recognition of preventive strategies along with therapeutic efficacy with minimal side effects is the new goal, thus turning from treatment to preventive approach [4].

Predominantly, the amyloid precursor protein (APP) is processed via the nonamyloidogenic pathway at the plasma membrane, where it is cleaved by alpha (α-secretase) within the Aβ domain, thus producing a soluble N-terminal fragment (sAPPα) and a membrane-bound C-terminal fragment, C83, which can be further cleaved by gamma (γ-secretases), generating p3 peptide, which thereby precludes the formation of Aβ. Importantly, A Disintegrin And Metalloproteinase (ADAM)10 has been identified as the constitutive α-secretase in the process of APP cleavage, playing a critical role in reducing the generation of Aβ peptides [5] and thus fostering neuroprotection [6]. Moreover, the soluble fragment sAPPα exerts beneficial physiological, biochemical, and behavioral effects along with augmenting neurogenesis [7]. Therefore, considering the distensible variety of ADAM10 substrates, natural compounds exhibiting the capacity to modulate and regulate ADAM10 levels are necessary [8]. APP is cleaved by β-secretase (BACE1, beta-site APP-cleaving enzyme 1), generating a soluble N-terminal fragment (sAPPβ) and a membrane-bound C-terminal fragment (C99) [9], which is subsequently cleaved by γ-secretase, releasing a cytoplasmic polypeptide termed AICD (APP intracellular domain), which functions as a transcription factor. β-secretase and γ-secretase are the key enzymes that catalyze the intramembrane proteolysis of APP pertaining to the amyloidogenic pathway [10,11]. The level of BACE1 and its activity are increased in AD brains; therefore, it plays a critical role in AD pathophysiology and is a prime target for regulating Aβ production in early AD [11], whereas presenilin-1 (PS1) encodes one of the major components of γ-secretase responsible for sequential proteolytic cleavage of APP. Significant elevated levels of PS1 have been observed in AD patients [12]. In pathological conditions, abnormal APP regulation causes excessive deposition of both the monomeric and oligomeric forms of neurotoxic Aβ peptides in the brain [13]. Taken together, α-secretase enhancers, β-secretase inhibitors, and/or γ-secretase inhibitors/modulators have been the prominent targets for AD therapeutics.

Tau is a microtubule-associated protein that, under pathological conditions, has the proclivity to aggregate into oligomers, paired helical filaments, and NFTs. Pathological tau conformations have also been shown to induce cognitive dysfunctions and bioenergetic impairments by affecting mitochondrial function [14]. Therefore, compounds with the capability of restoring normal tau homeostasis hold promise in preventing and/or treating the disease [15]. Additionally, glycogen synthase kinase-3β (GSK3-β) is a key player in AD pathophysiology since dysregulation of this kinase influences tau phosphorylation, Aβ production, memory, neurogenesis, and synaptic function [16]. Moreover, several studies have demonstrated that GSK3β is directly associated with both Aβ neurotoxicity and NFTs. In AD, excessive accumulation of Aβ can enhance the levels of GSK3β that contribute to the hyperphosphorylation of tau protein in neurons, subsequently leading to formation of NFTs [17,18,19].

Any disruption in the physiological homeostatic equilibrium leads to the development of disease followed by structural, metabolic, and functional changes, manifesting abnormal signs and symptoms [20]. As the explicit multifunctional physiological roles of Aβ and tau [21] have profound implications for AD pathology and therapeutics, it becomes extremely essential to maintain a balance contemplating both the physiological and pathological roles of Aβ and tau. Therefore, the idea of a homeostatic therapeutic approach, which is to prevent or treat diseases by modulating the homeostasis balance, is currently under implementation [22]. Furthermore, the complexity of AD pathogenesis necessitates the development of multi-target compounds that might aid in preventing and/or reversing the progression of AD by countering the dilatant pathological mechanisms like aberrant APP processing, toxic tau conformations, neuroinflammation, etc.

Huperzine A (Hup A) is a purified alkaloid compound extracted from a club moss called *Huperzia serrata*. With significant neuroprotective properties, several studies have documented both the cholinergic and non-cholinergic effects of this compound in context with AD pathology, which have been extensively reviewed [23]. Hup A effectively penetrates the BBB. Age-related changes in the brain result in blood–brain barrier (BBB) dysfunction and neurovascular damage. In this context, Hup A has been shown to prevent BBB dysfunction and neuroinflammation in rat hippocampus [24].

Given the therapeutic potential and toxicity parameters with minimal or no side effects, we investigated the role of Hup A on APP proteolysis and tau conformation under physiological conditions in human neuroblastoma SH-SY5Y cells. Molecular docking is an important component in the course of drug discovery. The molecular docking methods estimate the ligand–receptor binding free energy by evaluating critical phenomena involved in the intermolecular recognition with exploration of the ligand conformations adopted within the binding sites of molecular targets [25,26], Therefore, we performed molecular docking analysis in order to identify the binding sites of Hup A on α- and β-secretase. With an emphasis on the physiological dosage range, we propose the consideration of this natural compound as a homeostatic therapeutic agent that has the potential for both preventing and treating AD-like disorders.

## 2. Materials and Methods

### 2.1. Chemicals and Reagents

Minimum essential medium, Ham’s F-12 medium, fetal bovine serum, penicillin, and streptomycin were obtained from Gibco BRL (Gaithersburg, MD, USA). Huperzine A (Hup A) was purchased from Changsha Zhongren Biotechnology (Changsha City, China). For the primary antibodies, rabbit monoclonal anti-BACE1 and rabbit polyclonal anti-Aβ were purchased from Abcam (Cambridge, UK). Rabbit polyclonal anti-ADAM10, rabbit polyclonal anti-PS1, and rabbit monoclonal anti-p-GSK3β were purchased from Cell Signaling (Danvers, MA, USA); mouse monoclonal anti-p-tau and anti-GSK3β were purchased from Santa Cruz Biotechnology (Dallas, TX, USA), whereas mouse monoclonal anti-tau and mouse monoclonal β-actin were purchased from Millipore (Bedford, MA, USA); mouse monoclonal anti-APP-C99 and rabbit polyclonal anti-APP-C83 were purchased from Sigma-Aldrich (St. Louis, MO, USA). The secondary antibodies, rabbit polyclonal goat anti-rabbit IgG, and mouse polyclonal goat anti-mouse IgG were purchased from Sigma-Aldrich (St. Louis, MO, USA). ECL Prime Western Blotting Reagent^®^ was purchased from GE Healthcare (Little Chalfont, UK).

### 2.2. Cell Culture and Treatment

Human neuroblastoma SH-SY5Y cells were purchased from American Type Culture Collection (Manassas, VA, USA), and grown in complete medium composed of Eagle’s MEM and F12 (Nutrient Mixture F-12 (Ham’s), Gibco BRL (Gaithersburg, MD, USA)) in a 1:1 ratio. Hup A was prepared before each experiment by diluting in 10% DMSO, with the final concentration of DMSO being 0.01%. For the experiments, cells (1.5 × 10^6^ cells/well) were seeded in six-well plates and treated with serum-free media containing varying concentrations of Hup A (0, 0.1, 1, and 10 µM) for 24 h.

### 2.3. Western Blotting Analysis

Protein expression levels in each group were determined by Western blotting analysis. After treating with Hup A (0, 0.1, 1, and 10 µM) for 24 h, cells were lysed in RIPA buffer. SDS–PAGE was performed by loading samples into 10%, 15%, or 17.5% gels. Then, the protein bands were transferred to polyvinylidene difluoride membranes by electrophoresis, and subsequently, the membranes were blocked with either 3% bovine serum albumin or 3% nonfat dry milk in Tris buffer saline. Primary antibodies were added. Following an overnight incubation at 4 °C, the membranes were incubated with horseradish-conjugated anti-rabbit or anti-mouse IgG secondary antibodies for 90 min. ECL Prime reagents were applied to the membranes and visualized by chemiluminescence imaging and gel doc systems (G:BOX Chemi-XX6, Syngene, Merck, Cambridge, UK). Protein bands of interest were quantitatively analyzed using ImageJ^®^ software, version 1.8.0 (National Institutes of Health, Bethesda, MD, USA), normalized to β-actin bands.

### 2.4. Molecular Docking

The three-dimensional structures of BACE1 and ADAM10 were retrieved from the Protein Data Bank (PDB ID: 6EQM and 6BE6), and all nonstandard residues were removed. The missing residues of protein structures were fixed, and then, polar hydrogen atoms and charges were added. The direct interaction of Hup A with BACE1 and ADAM10 was investigated by molecular docking analysis using Autodock4 in the MGLTools suite [27]. The best ligand conformation with the lowest binding energy was searched using the Lamarckian genetic algorithm with predefined grid parameters. For BACE1, the grid was designed to cover its active site (grid center: *x* = 27.68 Å, *y* = 74.41 Å and *z* = 19.23 Å; grid dimension: *x* = 40.00 Å, *y* = 40.00 Å, *z* = 40.00 Å), while the grid parameters of ADAM10 covered the entire protein surface (grid center: *x* = 35.75 Å, *y* = 18.05 Å and *z* = 46.61 Å; grid dimension: *x* = 74.00 Å, *y* = 48.00 Å, *z* = 68.00 Å). The different binding modes were ranked as a cluster based on ligand conformation and the binding free energy (∆Gbinding: kcal/mol). The ligand poses with the lowest binding free energies were selected for writing protein–ligand complexes. Further molecular interaction analysis was performed using DS visualizer (Dassault Systèmes BIOVIA, 2016).

### 2.5. Enzymatic Activity Assay

Activity assays for BACE1 (β-secretase) and ADAM10 (α-secretase) were carried out according to the manufacturer’s instructions (AnaSpec, Fremont, CA, USA). The final concentrations of Hup A were 0.1, 1, and 10 μM. Inhibitors of BACE1 (LY2886721) or ADAM10 (GM 6001) obtained from the assay kits were employed for the experimental positive group (positive control). The fluorescence intensity was measured in a Synergy HTX multimode reader (BioTek, Winooski, VT, USA) at excitation/emission wavelengths of 490/520 nm.

### 2.6. Immunocytochemistry

Prior to cell plating, glass coverslips were placed in 24-well plates and then coated with poly-L-lysine. Cells at 80% confluence were treated with 10 µM Hup A for 24 h. After completion of the treatment period, cells were washed with phosphate-buffered saline (PBS) and fixed with 4% paraformaldehyde. A blocking solution consisting of 5% normal goat serum and 0.1% Triton X-100 in PBS were added, and cells were incubated in the solution for 1 h, then with rabbit anti-BACE1 (1:200) for another 1 h, followed by overnight incubation at 4 °C. Subsequently, cells were washed with PBS and incubated with Alexa 488-conjugated anti-rabbit (1:400) for 1 h and washed again. An antifade reagent (Vectashield, Vector Laboratories, Burlingame, CA, USA) was mounted onto the cells before visualization. Finally, cells were visualized using a FLUOVIEW FV3000 confocal laser-scanning microscope (Olympus, Tokyo, Japan).

### 2.7. Statistical Analysis

Data were processed using SPSS 22.0 (Statistical Package for Social Sciences, Chicago, IL, USA) for Windows and expressed as the mean  ±  standard error of the mean (SEM). One-way ANOVA and a post hoc least square difference (LSD) test were used to analyze the differences between each group. *p* values < 0.05 were considered statistically significant.

## 3. Results

### 3.1. Effects of Hup A on the Amyloidogenic Pathway

**Figure 1:**
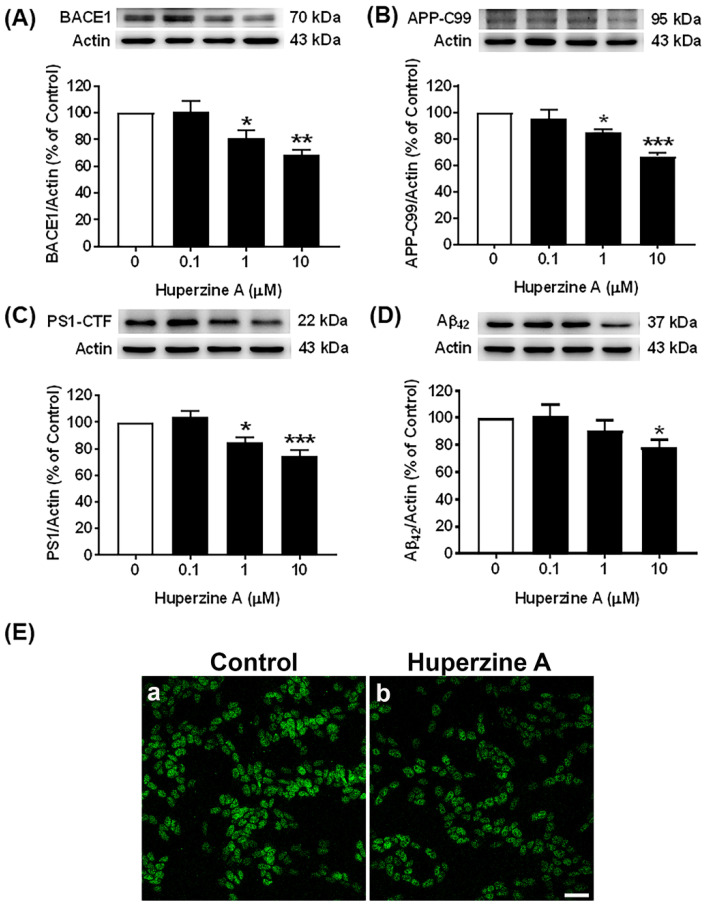
Effect of huperzine A (Hup A) on the amyloidogenic pathway in SH-SY5Y cells. Cells were treated with various concentrations of Hup A (0, 0.1, 1, and 10 µM) for 24 h. The levels of β-site amyloid precursor protein cleaving enzyme 1 (BACE1), a 99 amino acid C-terminal fragment of amyloid beta precursor protein (APP-C99), presenilin-1 (PS1), and amyloid beta 42 (Aβ42) protein were assessed using Western blot analysis. (**A**) Effect of Hup A on the levels of BACE1 protein. (**B**) Effect of Hup A on the levels of APP-C99 protein. (**C**) Effect of Hup A on the levels of the carboxy-terminal fragment of PS1 (PS1-CTF) protein. (**D**) Effect of Hup A on the levels of Aβ42 protein. (**E**) Confocal image of BACE1 immunostaining was captured after incubation in culture medium for 24 h. (**a**) Basal expression in the control cells. (**b**) Hup A-treated cells stained with anti-BACE1. Scale bar = 20 µm (20× magnification). Data are expressed as a percentage of the control and represented as the mean ± standard error of the mean (*n* = 4). Statistical significance was calculated using one-way ANOVA analysis (*, **, and *** denote the statistical significance at *p* < 0.05, *p* < 0.01, and *p* < 0.001, respectively, compared to the control group). (white bar = control and black bar = Hup A).

Our results showed that Hup A reduced the levels of both β- and γ-secretases (BACE1 and PS1) and subsequently decreased the levels of APP-C99 and Aβ_42_ oligomers compared with the control group, as shown in Figure 1A, Figure 1C, Figure 1B, and Figure 1D, respectively, Supplementary Figure S1. The protein levels of BACE1, PS1, and APP-C99 were significantly decreased by Hup A treatment at concentrations of 1 and 10 µM (Figure 1A–C), while the levels of Aβ_42_ oligomers were significantly reduced by the highest dose (10 µM) of Hup A treatment, suggesting an inhibitory effect of Hup A on Aβ_42_ production. In addition, the highest dose of Hup A at the concentration of 10 µM had the maximal effect to significantly reduce the protein levels of BACE1 (69.0 ± 3.4%, *p* < 0.01), PS1 (74.7 ± 4.3%, *p* < 0.001), APP-C99 (66.9 ± 2.3%, *p* < 0.001), and Aβ_42_ (78.3 ± 5.2%, *p* < 0.05) compared with the control group. We further confirmed the effect of Hup A on BACE1 expression by using immunocytochemistry. After treating with 10 µM Hup A for 24 h, BACE1 expression (Figure 1E(b)) slightly decreased, as depicted by green color, compared with the control group, respectively (Figure 1E(a)). Therefore, the present results indicate that Hup A can inhibit the amyloidogenic processing of APP by lowering the protein expression of BACE1 and PS1, further downregulating the protein levels of APP-C99 and Aβ_42_, respectively.

### 3.2. Effects of Hup A on the Nonamyloidogenic Pathway

**Figure 2:**
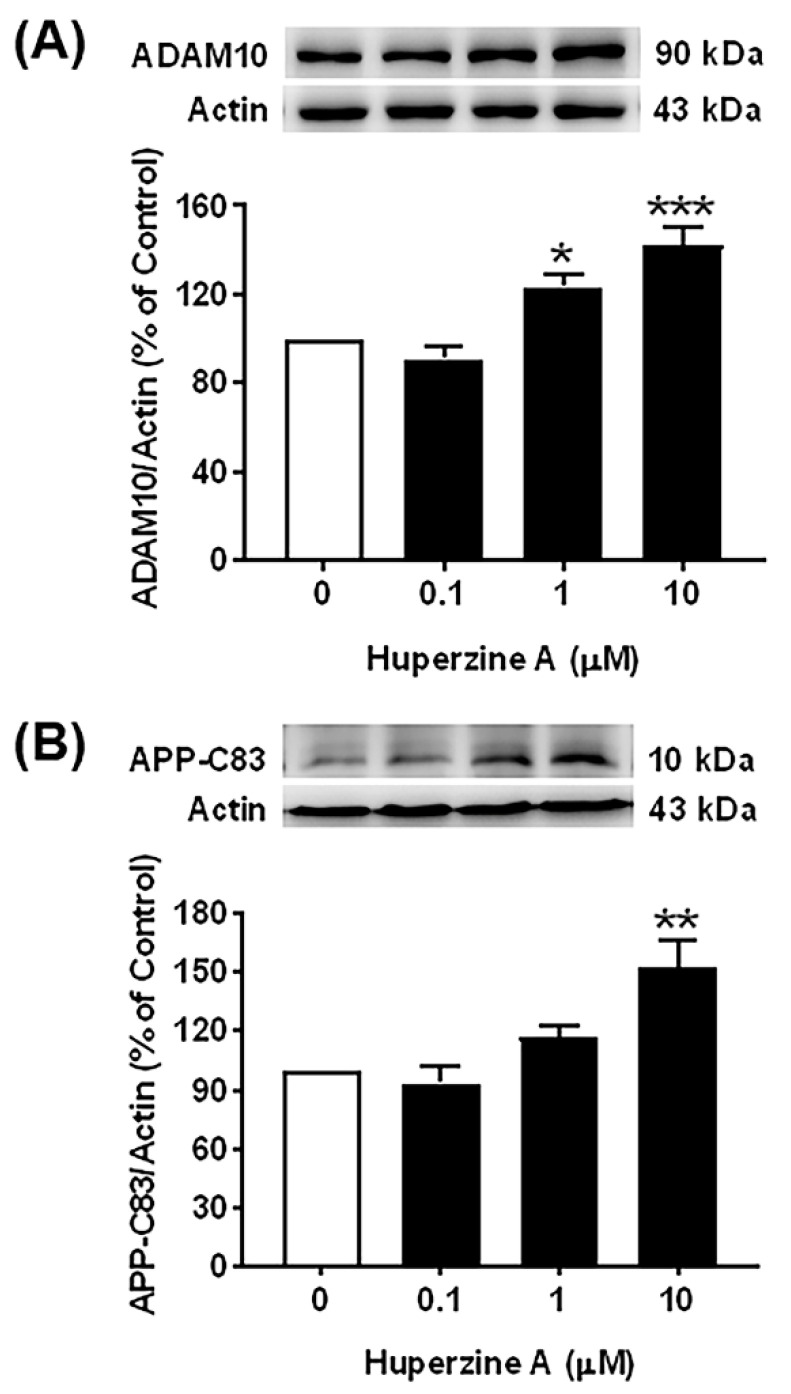
Effect of Huperzine A (Hup A) on the nonamyloidogenic pathway in SH-SY5Y cells. Cells were treated with various concentrations of Hup A (0, 0.1, 1, and 10 µM) for 24 h. The levels of a disintegrin and metalloproteinase 10 (ADAM10) and an 83 amino acid C-terminal fragment of amyloid beta precursor protein (APP-C83) were assessed using Western blot analysis. (**A**) Effect of Hup A on the levels of ADAM10 protein. (**B**) Effect of Hup A on the levels of APP-C83 protein. Data are expressed as a percentage of the control and represented as the mean ± standard error of the mean (*n* = 4). Statistical significance was calculated using one-way ANOVA analysis *, **, and *** denote the statistical significance at *p* < 0.05, *p* < 0.01, and *p* < 0.001, respectively, compared to the control group). (white bar = control and black bar = Hup A).

Our results demonstrated that Hup A enhanced the levels of α-secretase as well as ADAM10 protein and its intracellular product, APP-C83, as demonstrated in Figure 2A,B, Supplementary Figure S2. The protein levels of ADAM10 and APP-C83 were significantly increased after Hup A treatment at concentrations of 1 and 10 µM, respectively, compared with the control group. Moreover, the most effective dose of Hup A that could maximally increase the protein levels of ADAM10 (142.2 ± 8.0%, *p* < 0.001) and APP-C83 (153.2 ± 13.5%, *p* < 0.01) was 10 µM. Hence, these results suggest that Hup A can modulate the APP processing towards the nonamyloidogenic pathway by enhancing the protein expression of ADAM10 and APP-C83.

### 3.3. Regulation of BACE1 and ADAM10 Activity by Hup A

**Figure 3:**
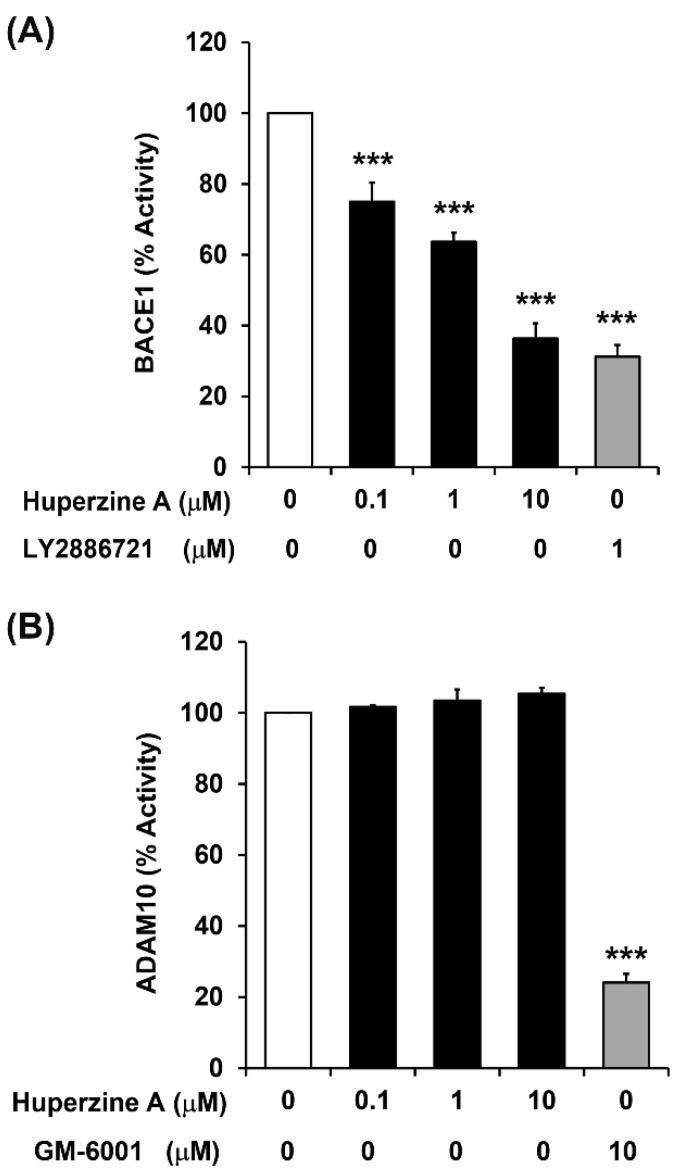
Effect of huperzine A (Hup A) on β-site amyloid precursor protein cleaving enzyme 1 (BACE1) and a disintegrin and metalloproteinase 10 (ADAM10) activity. The activity was assayed for α- or β-secretase activity as described in the section of materials and methods. (**A**) BACE1 catalytic activity. (**B**) ADAM10 catalytic activity. The results are expressed as a percentage of the control (white bar represents no drug treatment; dark bar represents different concentration of Hup A treatment; grey bar represents 1 µM LY2886721, the BACE1-specific inhibitor, or 10 µM GM6001, the ADAM10-specific inhibitor). The values in the bar graph represent the mean ± standard error of the mean of the specific fluorescence recorded during independent experiment (*n* = 4). Statistical analysis was performed using one-way ANOVA analysis (*** denotes statistical significance at *p* < 0.001 compared with untreated control). (white bar = control, black bar = Hup A, and grey bar = positive control).

The effects of Hup A on regulating the enzymatic activity of BACE1 and ADAM10 were investigated by treatment with final concentrations of 0.1, 1, and 10 μM. The results showed that Hup A significantly suppressed BACE1 activity in all treatment groups (Figure 3A). Notably, Hup A at a concentration of 10 μM was capable of inhibiting BACE1 enzymatic activity to the level observed in the group treated with the selective BACE1 inhibitor (1 μM LY2886721). In contrast, Hup A at final concentrations of 0.1, 1, and 10 μM had no significant effect on modulating ADAM10 activity (Figure 3B). GM 6001, a broad and potent matrix metalloprotease inhibitor, was used for the positive control and was found to significantly decrease ADAM10 activity at 10 μM. Taken together, the results indicate that Hup A is indeed a potent inhibitor of BACE1 activity but not a modulator of ADAM10 activity.

### 3.4. Direct Interactions of Hup A with the Active Site of BACE1/ADAM10

**Figure 4:**
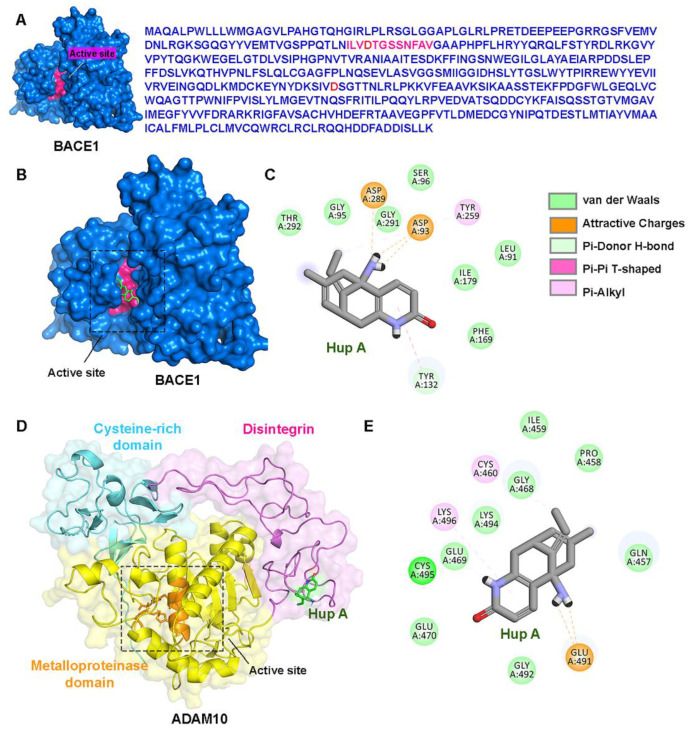
Molecular docking study of huperzine A (Hup A) and β-site amyloid precursor protein cleaving enzyme 1 (BACE1)/a disintegrin and metalloproteinase 10 (ADAM10) interactions. (**A**) The active site and protein structure of BACE1 are shown. The active site of BACE1 constitutes amino acids residues 90–101 (pink) and two aspartic residues (Asp93 and Asp and amino acids 289). (**B**) The best binding position of Hup A on BACE1 was predicted to be at the active site. (**C**) The 2D diagram shows amino acid residues lining the active site pocket interacting with Hup A. (**D**) The most favorable binding position of Hup A on ADAM10 was predicted at disintegrin domain (pink ribbons). (**E**) The interacting residues involved in Hup A interaction are illustrated using the 2D plot. (**C**,**D**) Van der Waals, attractive charges, and pi–pi interaction are indicated by green, orange, and pink dashed lines, respectively. The molecular structure of Hup A is shown as sticks with carbon, hydrogen, oxygen, and nitrogen atoms labelled with grey, white, red, and blue, respectively.

By using an in silico molecular docking analysis (Figure 4), we found that Hup A bound directly to the active site of BACE1, while it was assumed to bind to the ADAM10 disintegrin domain, which is distant from the active site. The most favorable binding free energies, i.e., ∆Gbinding, of Hup A on BACE1 and ADAM10 were −6.98 kcal/mol and −5.47 kcal/mol, respectively. The active site of BACE1 mainly comprises of residues 90–101, forming the substrate binding pocket. Hup A was shown to form salt bridges with the two active site aspartic residues (Asp93 and Asp289) and had hydrophobic interactions with other residues in the active site, including Leu91, Gly95, and Ser96 (Figure 4A–C). The H-bonds and ionic interactions of Hup A with Asp93 and Asp289 might be the key molecular elements responsible for the inhibitory effect of Hup A on BACE1 activity. In contrast, the molecular docking study showed that Hup A did not directly interact with the catalytic site of ADAM10. The best binding scores were found at the disintegrin domain of ADAM10 (Figure 4D,E). Therefore, this finding was consistent with the lack of an observed alteration in ADAM10 activity upon Hup A administration (Figure 3B)

### 3.5. Effect of Hup A on the GSK3β and Tau Pathways

**Figure 5:**
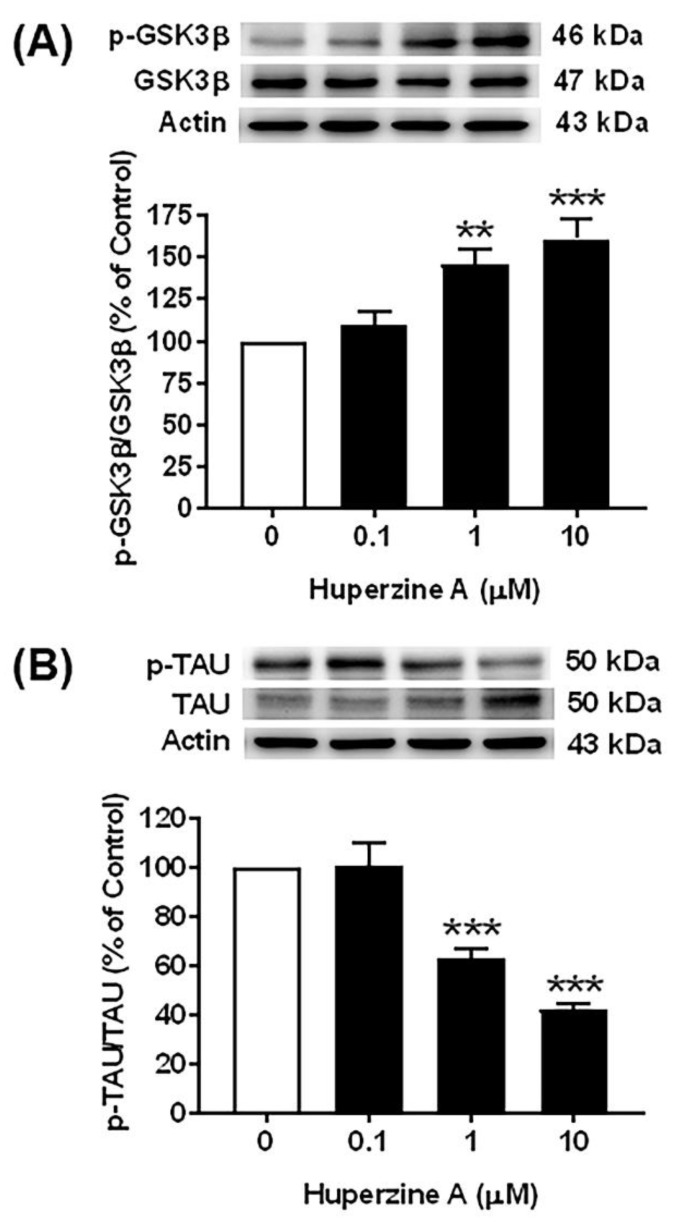
Effect of huperzine A (Hup A) on the glycogen synthase kinase-3 beta (GSK3β and tau pathways in SH-SY5Y cells. Cells were treated with various concentrations of Hup A (0, 0.1, 1, and 10 µM) for 24 h. The levels of phosphorylation of glycogen synthase kinase-3 beta (p-GSK3β), GSK3β, phosphorylation of tau (p-TAU), and total tau (TAU) protein were assessed using Western blot analysis. (**A**) Effect of Hup A on the ratio of the phosphorylated GSK3β to the total GSK3β (p-GSK3β/GSK3β). (**B**) Effect of Hup A on the ratio of phosphorylated tau to total tau protein (p-TAU/TAU). Data are expressed as a percentage of the control and represented as the mean ± standard error of the mean (*n* = 4). Statistical significance was calculated using one-way ANOVA analysis (** and *** denote the statistical significance at *p* < 0.01 and *p* < 0.001, respectively, compared to the control group). (white bar = control, black bar = Hup A).

The results showed that the levels of phosphorylated GSK3β (p-GSK3β), an inactive form of GSK3β that is phosphorylated at Ser-9, were significantly increased, whereas the levels of the total form of GSK3β were not changed after Hup A treatment at concentrations of 1 and 10 µM for 24 h compared with the control group, as illustrated in Figure 5A, Supplementary Figure S3. Then, the ratio of the phosphorylated GSK3β to the total GSK3β (p-GSK3β/GSK3β) was significantly increased after Hup A treatment at concentrations of 1 and 10 µM, as shown in Figure 5A. In addition, the most effective dose of Hup A was observed was of 10 µM, which resulted in a maximal increase in the ratio of p-GSK3β/GSK3β (160.9 ± 12.2%, *p* < 0.001). Therefore, the results indicate that Hup A robustly increases the levels of phosphorylated GSK3β-Ser-9, an inactive form of the GSK3β protein, suggesting that it might help in preventing tau phosphorylation. As expected, Hup A treatment at concentrations of 1 and 10 µM significantly mitigated the protein levels of phosphorylated tau (p-tau) compared with the control group, as shown in Figure 5B. On the other hand, the protein levels of total tau were significantly elevated by Hup A treatment at concentrations of 1 and 10 µM compared with the control group. Consequently, the ratio of phosphorylated tau to total tau protein (pTAU/TAU) was significantly attenuated after Hup A treatment at concentrations of 1 and 10 µM (Figure 5B). Moreover, the highest concentration (10 µM) of Hup A exerted the maximal effect to significantly lower the ratio of p-TAU/TAU (42.5 ± 2.1%, *p* < 0.001) in SH-SY5Y neuroblastoma cells. Thus, the results demonstrate that the protein levels of p-tau and p-tau/tau are lowered by Hup A treatment, suggesting the inhibitory effect of Hup A on tau phosphorylation.

## 4. Discussion

Although substantial treatment strategies have been developed to combat AD pathogenesis, the efficacy remains unsatisfactory, which is the reason why natural compounds are gaining popularity as alternative medicine due to their cost effectiveness and minimal side effects. Importantly, the therapeutic aim is turning towards prevention rather than a curative approach. The amyloid plaques and neurofibrillary tangles (NFTs) are the two distinctive pathological hallmarks of AD, which are also observed in the aging human brain [28]. Importantly, loss of APP homeostatic regulation in the brain vasculature possibly results in pathological outcomes in aging and neurodegenerative disorders. Therefore, we investigated the effects of Hup A on these pathways under physiological conditions in SH-SY5Y cells in order to analyze the role of this natural compound in regulating and modulating the homeostatic balance of the aforementioned molecular pathways. Human neuroblastoma SH-SY5Y cells have been widely used as experimental model for assessing APP proteolysis under physiological condition [29] as well as studying tau physiology [30].

Previous studies have shown the effect of Hup A on APP processing pathways under pathological conditions like in human embryonic kidney 293 APP Swedish mutant cells [31,32] and in APPswe/PS1dE9 transgenic mice [33], where specific mutations are introduced in order to enhance the overall Aβ production. Upregulation of BACE1 expression has been observed in the CSF of AD patients, where its levels correlate with the phosphorylated tau levels [34,35]. In the present study, we first investigated the effect of Hup A treatment on the amyloidogenic processing of APP. Several recent evidence indicates that counteracting early APP-C-terminal fragments (CTFs) accumulation might represent relevant therapeutic interventions in AD [36], as alterations in their quantitative and qualitative productions might directly contribute to the neuronal toxicity observed in the early phases of the disease [37]. Moreover, due to the fact that characterization of endogenous and exogenous APP-CTFs has been performed in SH-SY5Y cells, we also evaluated their levels post Hup A administration. The data revealed that Hup A in physiological concentrations downregulated the levels of BACE1 protein with significant attenuation of APP-C99 protein levels. In this context, it was shown that APP-C99 accumulates in AD-affected brain as well as in AD-like mouse models and detected earlier than Aβ, which suggests that it could be an early marker in AD pathology [38]. Interestingly, APP-C99 was found to act as a mediator of cholesterol disturbances occurring in AD [39]. Additionally, we demonstrated that Hup A treatment inhibited BACE1 catalytic activity. γ-secretase certainly is a drug target for reducing amyloid load. Developing γ-secretase-targeting compounds, i.e., inhibitors/modulators, is unquestionably a crucial domain in the therapeutic regime against AD. In the present study, we observed that PS1 protein levels were decreased post Hup A treatment, further leading to a marked reduction in the Aβ42 levels, as evident by Western blot and immunostaining evaluations.

On the contrary, we investigated the effect of Hup A exposure on the nonamyloidogenic proteolysis of APP, which plays a vital role in neuroprotection and the maintenance of the brain’s normal function [40]. ADAM10 was identified as an AD biomarker candidate and is reduced in the CSF [41] and platelets of AD patients compared to cognitively healthy individuals [8,42,43]. Intriguingly, a previous study in HEK293 APPswe cells also revealed that Hup A treatment enhanced the levels of sAPPα in a dose-dependent manner [31,32]. In the present study, we showed that Hup A augmented the nonamyloidogenic processing of APP by enhancing the protein levels of ADAM10 and APP-C83. However, treatments with various concentrations (0.1–10 µM) of Hup A had no effect on ADAM10 catalytic activity despite the increase in protein expression and its derivatives. Regarding the underlying mechanism, it was previously proposed that Hup A might affect the nonamyloidogenic pathway via activating the Wnt/β-catenin signaling [44].

Several docking studies have been performed to investigate the interaction and binding sites of Hup A pertaining to acetylcholinesterase, NMDA antagonists, human serum albumin, and human transferrin [45,46,47,48]. To our knowledge, this is the first study to predict the binding sites of Hup A in BACE1. The computational molecular docking study showed that Hup A could directly interact with the active site aspartic residues, including Asp93 and Asp289, by forming both H-bonds and ionic interactions. These two residues play an important role in APP cleavage; therefore, the direct molecular interaction of Hup A with BACE1 active sites could be the possible mechanism leading to the inhibition of BACE1 activity. In addition, confocal images of BACE1 immunostaining also showed that BACE1 expression was diminished by Hup A treatment. Hup A attenuates both the protein expression and the catalytic activity of BACE1, which accounts for the decrement in the Aβ levels as observed in the present study. However, the molecular interaction analyses showed that Hup A did not directly bind to ADAM10 active sites.

Dysregulation of tau phosphorylation cause aggregation of abnormally phosphorylated tau inclusions that are formed throughout the brain, resulting in neuronal dysfunction and death, a characteristic feature observed in neurodegenerative disorders like AD [49]. Aberrant activation of GSK3β leads to significant alterations in the posttranslational modifications in tau protein. Moreover, this kinase appears to be an important molecular integrator of several signals in the context of APP. A deregulation of its expression or activity could be associated with early stages of AD development. In addition, elevated Aβ levels can induce the production of an active form of the GSK3β protein, which results in the upregulation of the phosphorylated form of the tau protein and NFTs associated with pathological conditions in AD [50]. In this context, we demonstrated that the levels of the phosphorylated form of the GSK3β, an inactive form of the GSK3β protein, were augmented by Hup A treatment. Subsequently, the phosphorylated form of the tau protein was abolished post Hup A treatment in SH-SY5Y cells. Our findings demonstrate that Hup A affects the GSK3β and tau pathways by downregulating the GSK3β activity and the phosphorylation of tau. A previous study demonstrated that 2JY-OBZ4 (a Hup A analog) reduced AD conditions by regulating multiple targets, including GSK-3β and tau pathways [51]. However, a recent study has revealed that a well-known GSK-3β inhibitor, either Tideglusib or lithium, was not successful for treating AD in clinical trials [52]. Overall, our results indicate that Hup A has the potential to regulate and modulate the APP processing and tau conformation under physiological conditions, with strong implementation for its therapeutic use in neurodegenerative disorders like AD.

## 5. Conclusions

**Figure 6:**
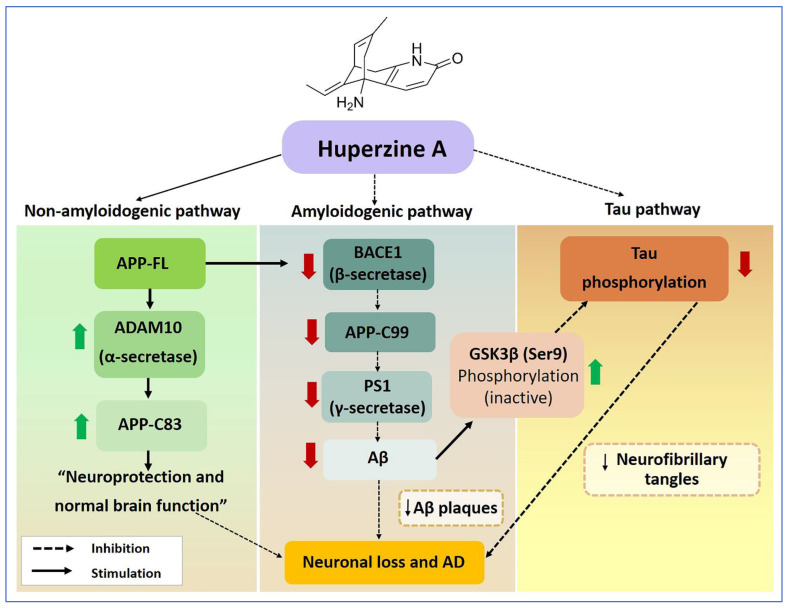
Schematic summary of the neuroprotective properties of huperzine A (Hup A). Hup A enhanced the expression of proteins in the nonamyloidogenic pathway by increasing the protein levels of a disintegrin and metalloproteinase 10 (ADAM10) and an 83 amino acid C-terminal fragment of amyloid beta precursor protein (APP-C83), which might help to improve neuronal loss and normal brain function. On the other hand, Hup A diminished the expression of proteins in the amyloidogenic pathway by lowering the levels of two major secretases, β-site amyloid precursor protein cleaving enzyme 1 (BACE1) and presenilin-1 (PS1), subsequently resulting in decreases in the production of both a 99 amino acid C-terminal fragment of amyloid beta precursor protein (APP-C99) and amyloid beta (Aβ). Moreover, Hup A increased the levels of phosphorylation of glycogen synthase kinase-3 beta (Serine9) (GSK3β (Ser9)), an inactive form of GSK3β protein, whereas it decreased the levels of phosphorylation of tau. Therefore, Hup A might help to reduce pathological events, including Aβ plaque and neurofibrillary tangle (NFT) formation, and relieve the progression of Alzheimer’s disease (AD). (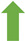 = increase, 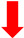 = decrease).

APP proteolytic processing is a highly attuned and controlled process. Therefore, imbalance in the normal physiological levels of APP and its derivatives or deregulation in its proteolytic processing could be one of the fundamental pre-amyloidogenic changes involved in the onset of AD. Concurrently, the onset and progression of cognitive impairment correlates with accumulation of tau. Therefore, therapeutics targeting secretases involved in the generation of Aβ have been considered one of the most important strategies to develop disease modifying treatment measures for AD. In this frame of reference, our present study signifies the use of Hup A as a potent modulator and regulator of APP proteolysis and tau conformation, where its physiological dosage range aids in maintaining a homeostatic balance between the APP cleaving secretases, thus favoring the nonamyloidogenic processing and preventing the amyloidogenic processing as well as the hyperphosphorylation of tau protein (Figure 6). Conclusively, Hup A not only represents an intriguing multi-target strategy in AD treatment but also holds promise in forestalling age-associated pathological neurodegenerative changes without any significant toxicity or side effects.

## Data Availability

Data are contained within the article.

## Supplementary Materials

The following supporting information can be downloaded at: https://www.mdpi.com/article/10.3390/biology13070518/s1, Figure S1. Full-length blots of Figure 1: Original photographs for the full-length blots at four independent experiments of each protein marker of Figure 1A–D; Figure S2. Full-length blots of Figure 2: Original photographs for the full length blots at four independent experiments of each protein marker of Figure 2A,B; Figure S3. Full-length blots of Figure 5: Original photographs for the full-length blots at four independent experiments of each protein marker of Figure 5A, Figure 5.

## Abbreviations

AD, Alzheimer’s disease; NFTs, neurofibrillary tangles; Hup A, huperzine A; APP, amyloid beta precursor protein; BACE1, β-site amyloid precursor protein cleaving enzyme 1; PS1, presenilin-1; APP-C99, α 99 amino acid C-terminal fragment of amyloid beta precursor protein; Aβ 42, amyloid beta 42; ADAM10, α disintegrin and metalloproteinase 10; APP-C83, an 83 amino acid C-terminal fragment of amyloid beta precursor protein; GSK3β, glycogen synthase kinase-3β.
