# Supplementary material for: Huperzine A Regulates the Physiological Homeostasis of Amyloid Precursor Protein Proteolysis and Tau Protein Conformation—A Computational and Experimental Investigation
Source: Biology (Basel). 2024 Jul 12;13(7):518. doi: 10.3390/biology13070518 (PMC11273828; doi:10.3390/biology13070518)
Supplement: Supplementary file 1 [file biology-13-00518-s001.zip › biology-3080718-supplementary.pdf]

Supplementary data to:

Huperzine A Regulates the Physiological Homeostasis of Amyloid Precursor Protein Proteolysis and tau Protein Conformation- A Computational and Experimental Investigation

Suwakon Wongjaikam [1,2], Chutikorn Nopparat [3], Parichart Boontem [1], Jiraporn Panmanee [4], Nopporn Thasana [5,6] Mayuri Shukla [1], Piyarat Govitrapong [1,*]

[1] Chulabhorn Graduate Institute, Chulabhorn Royal Academy, Bangkok, 10210, Thailand,

[2] Cell and Animal Model Unit, Institute of Nutrition, Mahidol University, Nakhonpathom, 73170, Thailand,

[3] Innovative Learning Center, Srinakharinwirot University, Sukhumvit 23, Bangkok, 10110, Thailand,

[4] Research Center for Neuroscience, Institute of Molecular Biosciences, Mahidol University, Nakhonpathom, 73170, Thailand,

[5] Program in Chemical Sciences, Chulabhorn Graduate Institute, Chulabhorn Royal Academy, Bangkok, 10210, Thailand;

[6] Laboratory of Medicinal Chemistry, Chulabhorn Research Institute, Bangkok, 10210, Thailand

* Corresponding author.

Piyarat Govitrapong, Chulabhorn Graduate Institute, Chulabhorn Royal Academy, Bangkok, 10210, Thailand.

E-mail address: piyarat@cgi.ac.th

Raw data of full length western blots

Figure S1. Full length western blots concerning **Figure 1.** Effects of huperzine A on amyloidogenic pathway in SH-SY5Y cells. Cells were treated with various concentrations of Hup A (0, 0.1, 1 and 10 µM) for 24 hours. The levels of BACE1, APP-C99, PS1, and $A\beta_{42}$ protein were assessed using western blot analysis. C= control, H= huperzine A, X= another sample

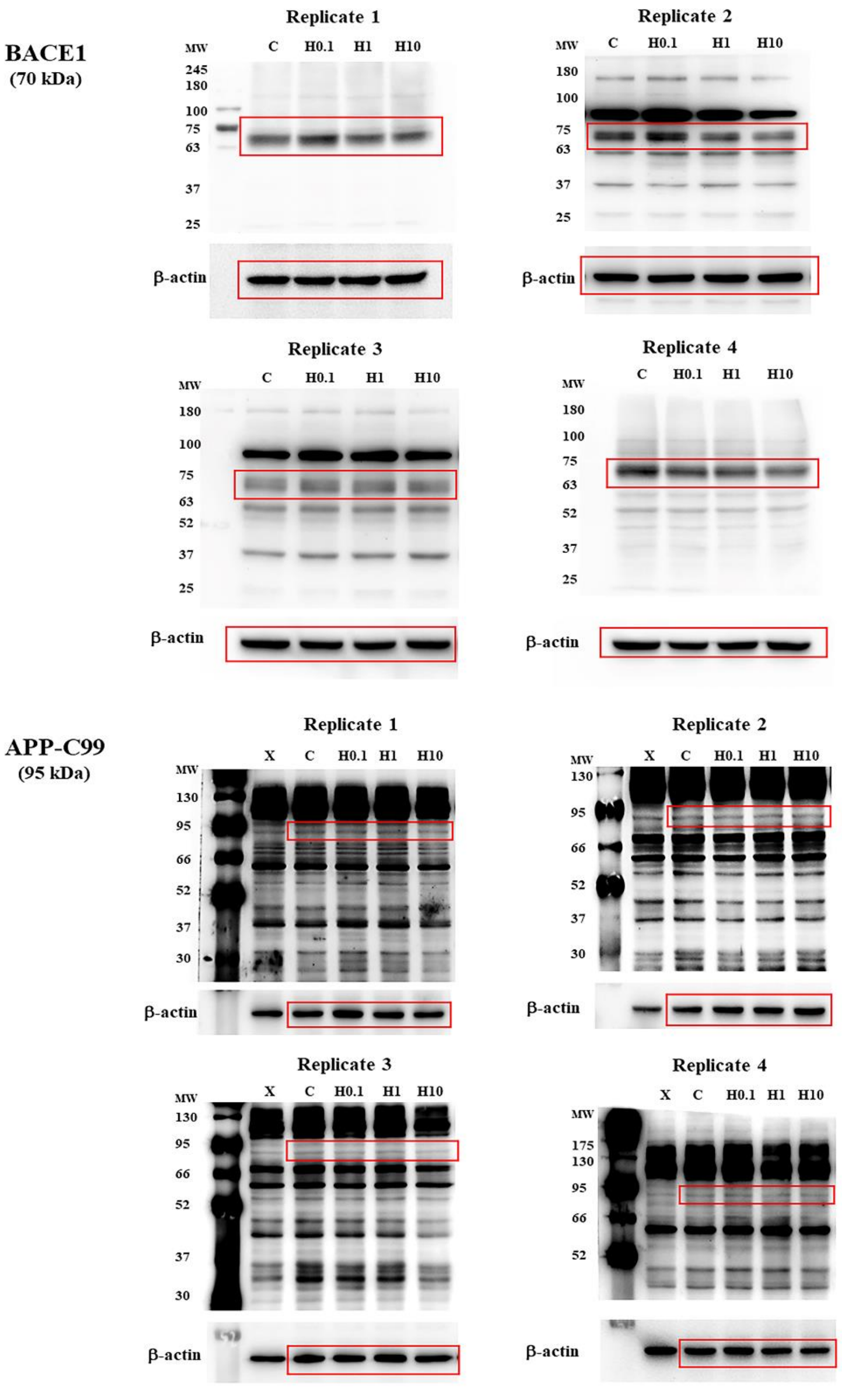

PS1-CTF
(22 kDa)

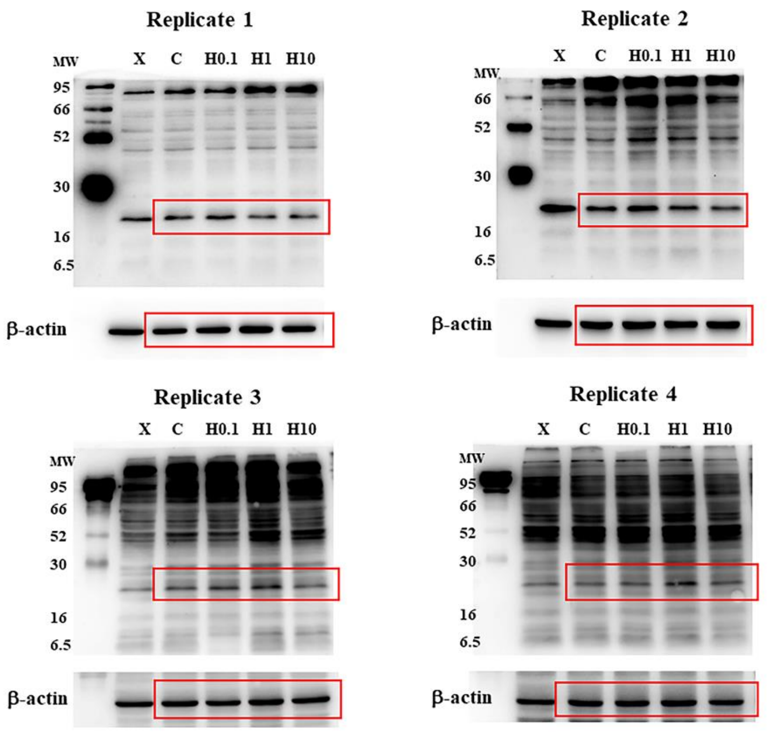

Aβ_{42}
(37 kDa)

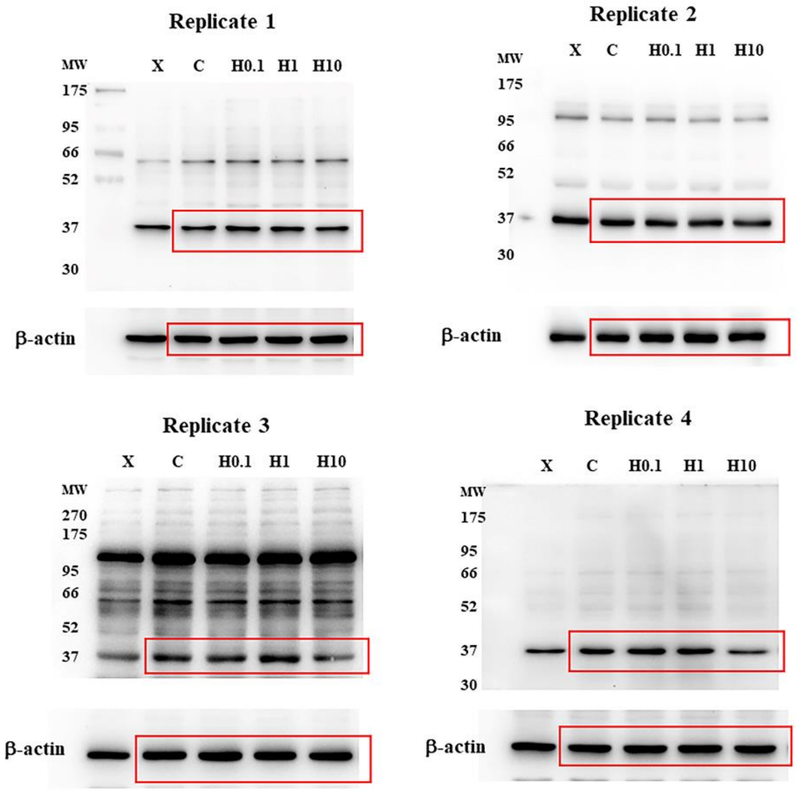

Figure S2. Full length western blots concerning **Figure 2.** Effects of huperzine A on non-amyloidogenic pathway in SH-SY5Y cells. Cells were treated with various concentrations of Hup A (0, 0.1, 1 and 10 µM) for 24 hours. The levels of ADAM10 and APP-C83 protein were assessed using western blot analysis. C= control, H= huperzine A, X= another sample

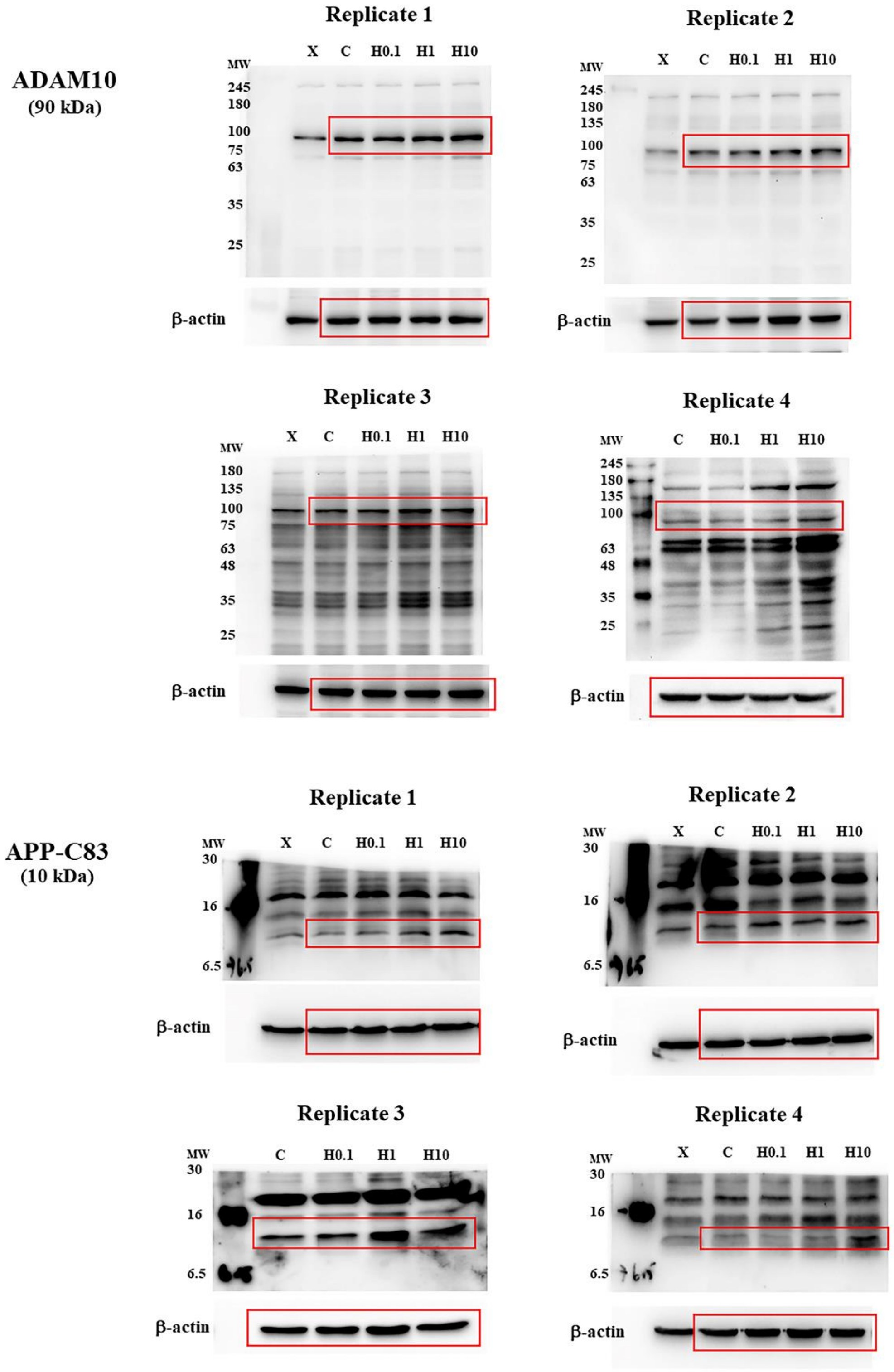

Figure S3. Full length western blots concerning **Figure 5.** Effects of huperzine A on GSK3β and tau pathway in SH-SY5Y cells. Cells were treated with various concentrations of Hup A (0, 0.1, 1 and 10 μM) for 24 hours. The levels of p-GSK3β, GSK3β, p-TAU and total tau (TAU) protein were assessed using western blot analysis. C= control, H= huperzine A, X= another sample

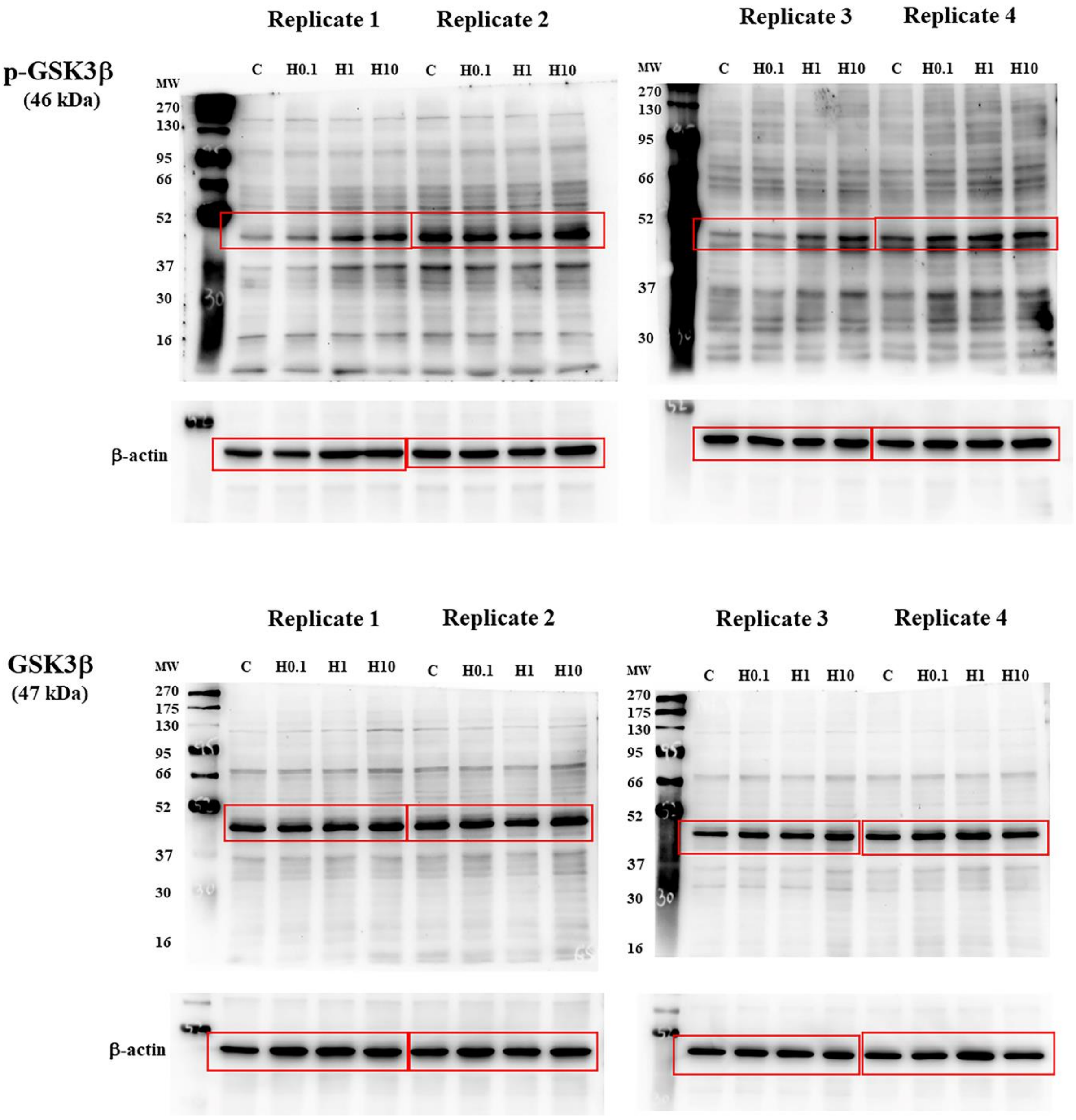

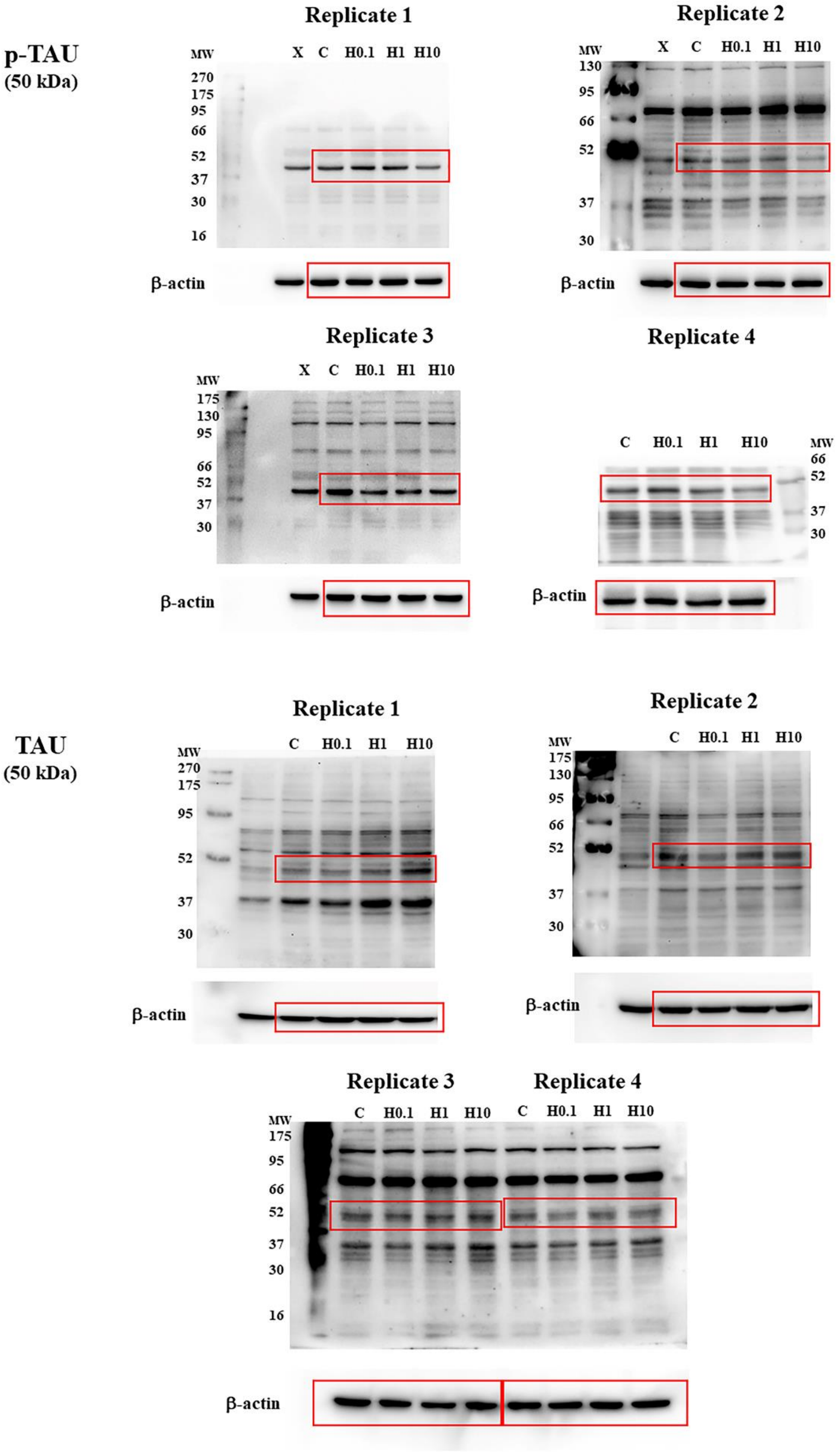
p-TAU
(50 kDa)
Replicate 1
MW
X C H0.1 H1 H10
270
175
95
66
52
37
30
16
β-actin
Replicate 2
MW
X C H0.1 H1 H10
130
95
66
52
37
30
β-actin
Replicate 3
MW
X C H0.1 H1 H10
175
130
95
66
52
37
30
β-actin
Replicate 4
C H0.1 H1 H10
MW
66
52
37
30
β-actin
TAU
(50 kDa)
Replicate 1
MW
C H0.1 H1 H10
270
175
95
52
37
30
β-actin
Replicate 2
MW
C H0.1 H1 H10
175
130
95
66
52
37
30
β-actin
Replicate 3
Replicate 4
MW
C H0.1 H1 H10
C H0.1 H1 H10
175
95
66
52
37
30
16
β-actin
